# Early Ambulation Following Lung Resection Surgery: Impact on Short-term Outcomes in Patients with Lung Cancer

**DOI:** 10.1298/ptr.E10277

**Published:** 2024-04-01

**Authors:** Kazunori KURATA, Yukio NAGATA, Keisuke OKI, Keishi ONO, Tomohiro MIYAKE, Kaori INUI, Masashi KOBAYASHI

**Affiliations:** ^1^Department of Rehabilitation, Kurashiki Central Hospital, Japan; ^2^Department of Thoracic Surgery, Kurashiki Central Hospital, Japan

**Keywords:** Lung Cancer, Perioperative, Pulmonary physical therapy, Postoperative pulmonary complications, Early ambulation

## Abstract

Objectives: Previous studies indicated that early ambulation following lung resection can prevent postoperative pulmonary complications (PPCs). However, some patients fail to achieve early ambulation owing to factors such as postoperative nausea, vomiting, or pain, particularly on postoperative day 1. This study aimed to address the critical clinical question: Is ambulation for ≥10 m during initial pulmonary rehabilitation necessary after lung resection surgery? Methods: This retrospective observational cohort study included 407 patients who underwent lung resection surgery for lung cancer between January 2021 and December 2022. Twelve patients with a performance status of ≥2 and 21 patients lacking pulmonary rehabilitation prescriptions were excluded. Patients were categorized into the “early ambulation” group, which included individuals ambulating ≥10 m during rehabilitation on the first postoperative day, and the “delayed ambulation” group. The primary outcome was PPC incidence, with secondary outcomes encompassing pleural drain duration, hospital length of stay, and Δ6-minute walk distance (Δ6MWD: postoperative 6MWD minus preoperative 6MWD). Results: The early and delayed ambulation groups comprised 315 and 59 patients, respectively. Significant disparities were noted in the length of hospital stay (7 [6–9] days vs. 8 [6–11] days, P = 0.01), pleural drainage duration (4 [3–5] days vs. 4 [3–6] days, P = 0.02), and Δ6MWD (−70 m vs. −100 m, P = 0.04). However, no significant difference was observed in PPC incidence (20.6% vs. 32.2%, P = 0.06). Conclusions: Ambulation for ≥10 m during initial pulmonary rehabilitation after lung resection surgery may yield short-term benefits as evidenced by improvements in various outcomes. However, it may not significantly affect the PPC incidence.

## Introduction

Lung cancer is a prominent global cause of mortality, and lung resection surgery represents a curative treatment modality. Preoperative pulmonary rehabilitation plays a pivotal role in mitigating the risk of postoperative pulmonary complications (PPCs), such as pneumonia, atelectasis, prolonged mechanical ventilation, and pulmonary fistula. PPCs increase the length of hospital stay after surgery, intensive care unit admissions, hospital readmissions, and mortality rates by 67%^1)^. Additionally, preoperative pulmonary rehabilitation contributes to improving 6-minute walk distances by 18–63 m and reducing the time until chest drain removal by 3–5 days^1,2)^. Postoperative pulmonary rehabilitation in patients undergoing lung resection surgery enhances exercise capacity and respiratory function, reduces fatigue, and improves health-related quality of life^3,4)^. Therefore, pulmonary rehabilitation plays a crucial role in the management of patients undergoing lung resection. Two multicenter investigations revealed that ≥90% of patients indicated for lung resection surgery underwent perioperative pulmonary rehabilitation^5,6)^. Jonsson et al.^7)^ observed improvements in physical activity due to perioperative pulmonary rehabilitation.

Early ambulation is an integral component of perioperative pulmonary rehabilitation. Ding et al. demonstrated that the first ambulation post-thoracoscopic lung surgery typically occurred after an average of 34.2 h. Their study found that patients who ambulated within 24 h experienced effective reductions in the length of hospital stay, PPCs, and chest drain tube duration^8)^.

However, some patients do not achieve early ambulation due to postoperative nausea and vomiting (PONV) or pain, particularly on the first day after surgery. Despite the relatively younger patient age in the study by Ding et al. (mean age, 54 years), only 45% managed ambulation within 24 h. Some studies have reported that perioperative pulmonary rehabilitation does not significantly reduce PPCs, nor does it lead to improvements in the length of hospital stay, exercise capacity, or health-related quality of life^9,10)^. Given that most lung resection patients in Japan are ≥65 years owing to an aging society, understanding the impact of early ambulation is crucial for this demographic.

This study aimed to address the following clinical questions: Is ambulation for ≥10 m during initial pulmonary rehabilitation necessary after lung resection surgery? Does it affect outcomes in older patients?

## Methods

### Study design, patients, and setting

This retrospective observational cohort study included 407 patients diagnosed with lung cancer who were admitted to our institution for resection surgery between January 1, 2021, and December 31, 2022. Twelve patients with the Eastern Co-operative Oncology Group performance status (PS) scores^11)^ of 2, 3, or 4 and 21 patients without a rehabilitation prescription were excluded. In cases where a patient underwent multiple lung resection surgeries during the study period, data from their initial surgery were utilized. Patients were categorized into two groups: the early ambulation group (early group), comprising patients who ambulated ≥10 m during rehabilitation on the first postoperative day, and the “delayed ambulation” group (delayed group). We categorized the groups based on a 10-m threshold distance because we aimed to evaluate the effectiveness of pulmonary rehabilitation. Some patients were able to walk to a restroom located within their rooms, which was approximately 5 m away; however, they were unable to walk during the initial pulmonary rehabilitation sessions because of PONV or pain. Patients used a specialized cart designed for carrying a chest drain bag to assist with mobility during their ambulation. Furthermore, they received personalized support from a physical therapist, as deemed necessary.

Additionally, patients were subcategorized into two groups based on the extent of resection: the lobectomy group (including pneumonectomy and bilobectomy) and the segmentectomy/wedge resection group. The purpose of this subanalysis was to examine the effect of the extent of resection on outcomes.

This study was approved by the Institutional Review Board and the Ethics Committee for Clinical Studies of Kurashiki Central Hospital (IRB number: 4063) and conducted in compliance with the Declaration of Helsinki.

### Data collection

Data collected from medical records included age, sex, body mass index, diagnosis, lung cancer stage, medical history, smoking history, PS, surgical details (approach, extent of resection, duration of surgery), presurgery exercise capacity assessments (6-minute walk test [6MWT]), and presurgery respiratory function measurements (vital capacity predicted, forced expiratory volume in the 1st second/forced vital capacity, diffusing capacity for carbon monoxide predicted). Data from the first postoperative day (walking distance during pulmonary rehabilitation, PONV, pain evaluated by the modified Borg scale) as well as PPCs, chest tube duration, length of hospital stay after surgery, and postsurgery 6MWT results were collected from the medical records.

### Outcomes

1. Incidence of PPCs

PPCs included pneumonia, atelectasis, prolonged mechanical ventilation, hypoxemia, and pulmonary fistulas. In accordance with the Clavien–Dindo classification^12)^, we included cases of pneumonia and atelectasis classified as Grade II or higher as well as cases of pulmonary fistula classified as Grade IIIa or higher. Hypoxemia was defined when patients required home-based oxygen therapy upon hospital discharge. We excluded cases of PPCs with onset prior to the initiation of pulmonary rehabilitation, because the primary focus of this study was to investigate the efficacy of early ambulation following surgery.

2. Duration of pleural drainage

Pleural drainage tubes were inserted during surgery and removed by the surgeon when the daily pleural fluid output reached <200 cc. The duration, measured in days from insertion to removal, was used as an indicator of pleural drainage duration.

3. Length of hospital stay after surgery

Some patients were admitted to the hospital earlier to manage their blood sugar levels or for antithrombotic drug regimens. The primary aim of this study was to assess the efficacy of early postoperative ambulation. Therefore, the number of days after surgery was used as a measure of length of hospital stay.

4. Postoperative exercise capacity

Δ6-minute walk distance (Δ6MWD) was calculated as the postoperative 6MWD minus the preoperative 6MWD. The 6MWT was performed by trained physical therapists^13)^.

### Statistical analysis

Continuous data were expressed as either mean ± standard deviation or median with interquartile range, while categorical data were expressed as numbers and percentages. Data normality was assessed using the Shapiro–Wilk test. Group differences for normally distributed continuous variables were evaluated using the Student’s t-test, while the Mann–Whitney U test was employed for continuous variables with skewed distributions. Differences in categorical variables were examined using the χ^2^ test. Statistical significance was set at P <0.05. All statistical analyses were conducted using IBM SPSS Statistics (version 20.0; IBM Armonk, NY, USA).

## Results

**Fig. 1.**
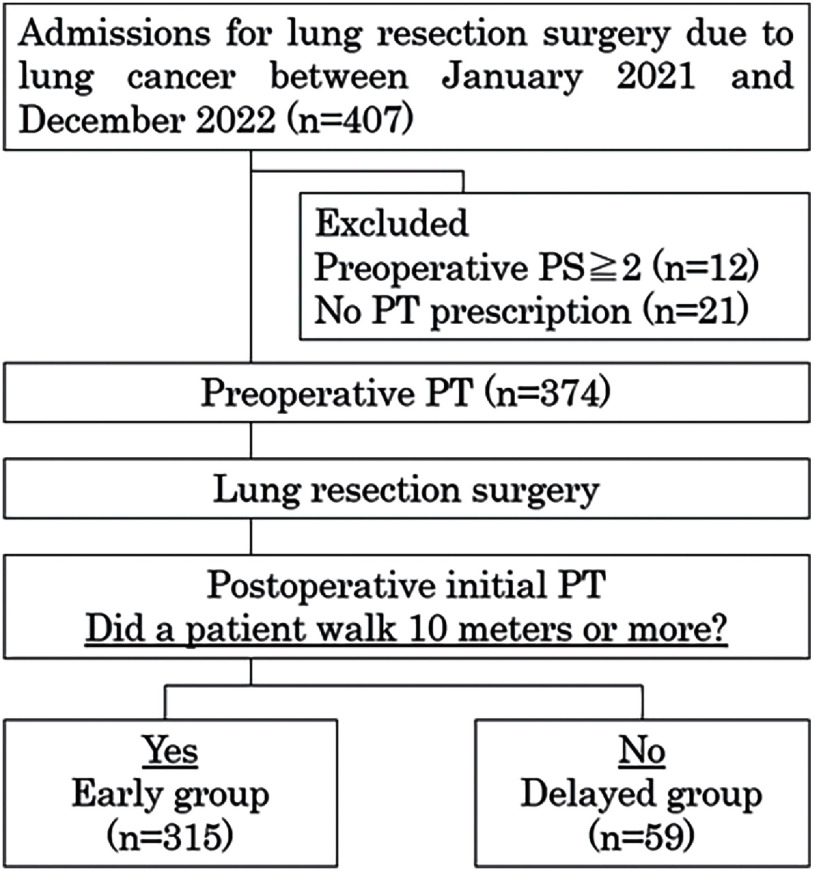
Study protocol PS: performance status; PT: physical therapy

A total of 374 patients were included in this study (**Fig. 1**). The clinical and demographic data are presented in **Table 1**. The median age was 73 years, and 65.5% of the patients were male. Seventy percent had adenocarcinoma and 22.7% had squamous cell carcinoma. The most common surgical approaches and extent of resection were video-assisted thoracoscopic surgery (VATS) (52.9%) and lobectomy (63.1%), respectively.

**Table 1.** Clinical characteristics of the patients

Variables	Total (n = 374)	Early group (n = 315)	Delayed group (n = 59)	P-value
Age, years [IQR]	73 [68–78]	73 [68–77]	75 [69–78]	0.36
Male gender, n (%)	245 (65.5)	213 (67.6)	32 (54.2)	0.053
BMI (kg/cm^2^), mean (SD)	23.0 (3.8)	23.2 (0.2)	22.2 (0.5)	0.97
Surgical approach, n (%)				
RATS	73 (19.5)	62 (19.7)	11 (18.6)	
VATS	198 (52.9)	171 (54.3)	27 (45.8)	
Thoracotomy	103 (27.5)	82 (26.0)	21 (35.6)	0.30
Extent of resection, n (%)				
Segmentectomy	82 (21.9)	69 (21.9)	13 (22.0)	
Lobectomy	236 (63.1)	199 (63.2)	37 (62.7)	
Bilobectomy	7 (1.9)	6 (1.9)	1 (1.7)	
Pneumonectomy	3 (0.8)	1 (0.3)	2 (3.4)	
Wedge resection	46 (12.3)	40 (12.7)	6 (10.2)	0.19
Pathological stage, n (%)				
Stage 0	20 (5.3)	17 (5.4)	3 (5.4)	
Stage 1	273 (73.0)	231 (73.3)	42 (71.2)	
Stage 2	53 (14.2)	46 (14.6)	7 (11.9)	
Stage 3	27 (7.2)	20 (6.4)	7 (11.9)	
Stage 4	1 (0.3)	1 (0.3)	0 (0)	0.63
Disease type, n (%)				
Adenocarcinoma	258 (70.0)	217 (68.9)	41 (69.5)	
Squamous	85 (22.7)	78 (24.8)	7 (11.9)	
Small cell	8 (2.1)	7 (2.2)	1 (1.7)	
Adenosquamous	8 (2.1)	5 (1.6)	3 (5.1)	
Large cell	3 (0.8)	3 (1.0)	0 (0)	
Others	12 (3.2)	5 (1.6)	7 (11.9)	0.14
Duration of operation, min. [IQR]	170 [129–221]	169.5 [128.3–216]	187 [134–264]	0.09
Blood loss, ml [IQR]	0 [0–50]	0 [0–50]	0 [0–62]	0.25
Preoperative 6MWT				
6MWD, m [IQR]	413 [360–465]	415 [360–465]	410 [360–455]	0.54
Min. SpO_2_, % [IQR]	95 [94–96]	95 [94–96]	95 [94–96]	0.34
Max. PR, bpm [IQR]	105 [96–116]	105 [96–117]	104.5 [94.8–114]	0.32
Mod. Borg scale, [IQR]	2 [1–4]	2 [1–4]	2 [1–3]	0.70
Performance status, n (%)				
0	272 (72.7)	235 (74.6)	37 (62.7)	
1	102 (27.3)	80 (25.4)	22 (37.3)	0.08
Respiratory function				
VC pred., % (SD)	105.4 (16.0)	105.6 (0.9)	104.7 (2.1)	0.64
FEV1/FVC, % [IQR]	70.6 [63.1–78.9]	70.3 [63.1–78.7]	71.5 [63.6–79.3]	0.50
DLCO pred., % [IQR]	95.5 [79.2–125.4]	95.3 [79.2–124.6]	99.4 [78.3–129.6]	0.57
Postoperative 6MWT				
6MWD, m [IQR]	332.5 [290–387.5]	337.5 [292.3–390]	320 [280–368.8]	0.18
Min. SpO_2_, % [IQR]	93 [91–95]	93 [91–95]	94 [91.3–95]	0.13
Max. PR, bpm [IQR]	104 [95–113]	104 [96–114]	102 [93–108]	0.14
Mod. Borg scale, [IQR]	3 [2–4]	3 [2–4]	3 [2–4]	0.50

IQR, interquartile range; BMI, body mass index; SD, standard deviation; RATS, robot-assisted thoracoscopic surgery; VATS, video-assisted thoracoscopic surgery; 6MWT, 6-minute walk test; 6MWD, 6-minute walk distance; SpO_2_, saturation of percutaneous oxygen; PR, pulse rate; VC, vital capacity; FEV_1_/FVC, forced expiratory volume in 1 second/forced vital capacity; DLCO, diffusing capacity of the lung carbon monoxide

The early and delayed groups consisted of 315 and 59 patients, respectively. No significant differences were observed between the two groups in terms of age, sex, surgical approach, extent of resection, pathological stage, disease type, duration of surgery, blood loss, preoperative 6MWT, pulmonary function, PS, or postoperative 6MWT.

**Table 2** presents the study outcomes. The overall incidence of PPCs among patients was 22.5%, of which 20.6% were in the early group and 32.2% were in the delayed group. The incidences of atelectasis, pneumonia, pulmonary fistula, and hypoxemia were 2.7% (2.5% in the early group vs. 3.4% in the delayed group), 2.9% (2.9% vs. 3.4%), 9.6% (9.2% vs. 11.9%), and 8.6% (7.9% vs. 11.9%), respectively. The median duration of pleural drainage was 4 days (4 days in the early group vs. 4 days in the delayed group, P = 0.02), median length of hospital stay after surgery was 7 days (7 days in the early group vs. 8 days in delayed group, P = 0.006), and the median Δ6MWD was −80 m (−70 m in the early group vs. −100 m in the delayed group, P = 0.04). Significant differences were observed between the two groups.

**Table 2.** Outcomes: incidence of PPCs, duration of pleural drainage, length of hospital stay, and Δ6MWD

Variables	Total(n = 374)	Early group(n = 315)	Delayed group (n = 59)	P-value	Statisticalpower
PPCs, n (%)	84 (22.5)	65 (20.6)	19 (32.2)	0.06	
Atelectasis	10 (2.7)	8 (2.5)	2 (3.4)	0.66	
Pneumonia	11 (2.9)	9 (2.9)	2 (3.4)	0.68	
Pulmonary fistula	36 (9.6)	29 (9.2)	7 (11.9)	0.47	
Hypoxemia	32 (8.6)	25 (7.9)	7 (11.9)	0.31	–
Duration of pleural drainage, days [IQR]	4 [3–5]	4 [3–5]	4 [3–6]	0.02	0.34
Length of hospital stay, days [IQR]	7 [6–9]	7 [6–9]	8 [6–11]	0.01	0.38
Δ6MWD, m [IQR]	80 [−120 to −27]	−70 [−120 to −25]	−100 [−133.8 to −52]	0.04	0.42

PPCs, postoperative pulmonary complications; IQR, interquartile range; Δ6MWD, Δ6-minute walk distance

**Table 3** describes the relationship between the extent of resection and the outcomes. The duration of pleural drainage (4 vs. 4) and length of hospital stay (7 vs. 6) were longer in the lobectomy group than those in the segmentectomy/wedge resection group. However, no significant differences were observed in the incidence of PPCs and Δ6MWD between the two groups.

**Table 3.** Extent of resection and outcomes

Variables	Lobectomy, bilobectomy, and pneumonectomy (n = 246)	Segmentectomy and wedge resection (n = 128)	P-value
PPCs, n (%)	61 (24.8)	23 (18.0)	0.15
Atelectasis	8 (3.3)	2 (1.6)	0.50
Pneumonia	10 (4.1)	1 (0.8)	0.11
Pulmonary fistula	27 (11.0)	9 (7.0)	0.27
Hypoxemia	20 (8.1)	12 (9.4)	0.70
Duration of pleural drainage, days [IQR]	4 [3–5]	4 [3–4]	<0.001
Length of hospital stay, days [IQR]	7 [6–9]	6 [5–9]	<0.01
Δ6MWD, m [IQR]	−80 [−120 to −24.5]	−80 [−120 to −30]	0.97

PPCs, postoperative pulmonary complications; IQR, interquartile range; Δ6MWD, Δ6-minute walk distance

**Table 4** describes the relationship between pain and PONV within the two studied groups. While pain showed no significant difference, PONV in the delayed group was significantly higher (61.0% vs. 24.4%, P <0.001).

**Table 4.** Pain and PONV of the first postoperative day

Variables	Total(n = 374)	Early group(n = 315)	Delayed group(n = 59)	P-value
PONV, n (%)				
Yes	113 (30.2)	77 (24.4)	36 (61.0)	
No	261 (69.8)	238 (75.6)	23 (39.0)	<0.001
Pain, numerical rating scale [IQR]	2 [0–3]	2 [0–3]	3 [0–4]	0.18

PONV, postoperative nausea and vomiting; IQR, interquartile range

## Discussion

The primary objectives of this study were to investigate the necessity of early ambulation during pulmonary rehabilitation following lung resection surgery and its impact on outcomes among older patients. Our findings indicate that early ambulation during pulmonary rehabilitation in older patients yields several advantages, including expedited removal of pleural drainage tubes, reduced hospitalization duration, and the maintenance of exercise capacity. Notably, while there were benefits observed, the incidence of PPCs did not exhibit a significant difference between the two groups. While many previous studies have exclusively enrolled patients with non-small cell lung cancer, our study extended the inclusion criteria to include both patients with small cell lung cancer and non-small cell lung cancer in order to examine the short-term outcomes, such as the incidence of PPCs, exercise capacity following surgery, length of hospital stay, and duration of pleural drainage, instead of focusing on long-term outcomes. Consistent with previous studies, 95% of the patients in our study were diagnosed with non-small cell lung cancer. Furthermore, 94.7% of the patients underwent pulmonary rehabilitation. This aligns with previous studies, which have consistently reported that ≥90% of the patients received physical therapy^5,6)^. While most studies^14)^ focused on patients aged 60–65 years, the median age of the patients included in our study was 73–75 years. Therefore, this study presents results specific to older patients.

### Incidence of PPCs

While our study showed no statistically significant difference in PPC incidence between the early and delayed ambulation groups, a study by Ding et al. in 2023 reported a significantly lower PPC incidence when ambulation was initiated within 24 h after surgery. The mean age in their study was 54–57 years, whereas that in our study was 73–74 years. This disparity in age may have influenced the timing of ambulation after surgery. Additionally, it is important to note that their study focused exclusively on VATS. The incidence of PPCs has been reported to range from 13% to 16.6% in lobectomy cases^15–17)^ and 2% in VATS^18)^. Given the existing data, we anticipated a lower PPC incidence in VATS cases. Our study’s broader procedural range, including various surgeries beyond VATS, added depth to the findings. Additionally, our study included patients who required home-based oxygen therapy upon discharge from the hospital. The choice of surgical approach and the definition of PPCs may have influenced our results. However, there were no significant differences in the incidence of pneumonia, atelectasis, pulmonary fistula, and hypoxemia between the two groups; however, all incidences were lower in the early group than those in the delayed group. This finding suggests that early ambulation may play a role in reducing PPCs. Several studies have reported the beneficial effects of preoperative pulmonary rehabilitation in reducing the incidence of PPCs^19,20)^. The combination of preoperative and perioperative pulmonary rehabilitation is of utmost importance.

### Duration of pleural drainage, length of hospital stay, and Δ6MWD

Our findings reinforce the benefits of early ambulation after lung resection surgery. Specifically, the duration of pleural drainage, length of hospital stay, and Δ6MWD were lesser for patients who practiced early ambulation than for those with delayed ambulation. These findings are comparable to Ding et al.’s study, which indicated that early ambulation within the first 24 h postoperatively led to improvements in both the duration of pleural drainage and hospital stay, corroborating the positive impact of early mobilization on postoperative recovery^8)^.

Pleural chest drainage has been identified as an inhibitory factor in early ambulation^21)^. Early removal of chest drains has been reported to promote early ambulation and reduce the incidence of PPCs^22)^. Despite the inability to quantify the pleural drainage volume, early ambulation may have a positive impact on improvement. Early mobilization could potentially enhance the efficiency of pleural drainage following surgery. Moreover, most older patients spend approximately 80% of their day in bed^23)^, and immobilization can result in a decrease of 1%–2% in skeletal muscle mass daily^24)^. Early removal of chest drains eliminates physical impediments, allowing smooth progression of ambulation. This, in turn, likely contributes to the reduction in postoperative hospitalization days and the maintenance of exercise tolerance. Moreover, retention of pleural drains has been suggested to decrease postoperative lung capacity^25)^. The early removal of drains in the early ambulation group may have contributed to the preservation of lung function. However, it is essential to note that the median time for drain removal in both groups in this study was 4 days, and the length of hospital stay was 7–8 days, indicating a marginal difference.

Finally, in Japan, lung cancer surgery is managed through insurance coverage and clinical pathways, making it challenging to observe significant differences in outcomes. Despite these limitations, our results underscore the importance of early ambulation during recovery.

### Impact of the extent of resection on outcomes

Our selected outcomes were suspected to be influenced by the extent of resection, prompting us to conduct a subanalysis. The subanalysis on the extent of resection indicated no significant difference in Δ6MWD, suggesting that pulmonary rehabilitation and early ambulation may aid in maintaining exercise capacity.

Previous studies have reported a higher chest tube output following lobectomy and bilobectomy than after segmentectomy and wedge resection^26)^, and prolonged pleural drainage has been linked to an extended length of hospital stay^27)^. Both early ambulation and the extent of resection contributed to patient outcomes.

### Early ambulation and PONV

The incidence of PONV was significantly higher in the delayed group. Pain had no effect on early ambulation during pulmonary rehabilitation; this was due to opioid use for pain control, which affected the incidence of PONV. Female sex, history of motion sickness or PONV, nonsmoking, and the use of postoperative opioids were reported as risk factors for PONV^28)^, and the incidence of PONV was significantly higher with lobectomy than that with VATS^29)^. Accurate identification of these risk factors may facilitate the promotion of postoperative ambulation on the following day, potentially enhancing the outcomes gleaned from this study.

### Limitations

This study had some limitations. First, this was a retrospective single-center investigation, and there were instances of missing data. Second, the incidence of PPCs was not defined after surgery but only after the initial pulmonary rehabilitation; defining PPCs after initial pulmonary rehabilitation might overlook cases where symptoms develop before therapy initiation. Third, we exclusively monitored physical activity during pulmonary rehabilitation and did not account for other activities such as sitting on a bed or chair or walking to a restroom. Some patients showed acceptable levels of physical activity even though they could not walk during pulmonary rehabilitation on the first day after surgery. Fourth, the results should be interpreted with caution because patients who could walk ≥10 m after surgery might have been healthier. Further studies are needed to elucidate the relationship between early ambulation and patient outcomes.

## Conclusions

Our findings suggest that ambulation for ≥10 m during initial pulmonary rehabilitation in older patients is associated with several benefits, including earlier removal of pleural drainage tubes, shortened hospital stay, and preservation of exercise capacity.

## Conflict of Interest

The authors have no conflicts of interest in relation to this manuscript.
